# Highly focused human CD8^+^ T-cell response in the lower airways during acute influenza infection

**DOI:** 10.1093/jimmun/vkag068

**Published:** 2026-05-19

**Authors:** Adam Saidu, Jeremy Chase Crawford, Sarah Walden, Jia-Hua Qu, Heather M Machkovech, Fangjie Han, Resha Sisti, E Kaitlynn Allen, Daniel Reynolds, Nicholas Borcherding, Jackson S Turner, Thomas C Friedrich, Derek E Byers, Ali H Ellebedy, Paul G Thomas, Philip A Mudd

**Affiliations:** Department of Emergency Medicine, Washington University School of Medicine, Saint Louis, MO, United States; Department of Host-Microbe Interactions, St. Jude Children’s Research Hospital, Memphis, TN, United States; Center for Infectious Diseases Research, St. Jude Children’s Research Hospital, Memphis, TN, United States; Department of Microbiology, Immunology and Biochemistry, University of Tennessee Health Science Center, Memphis, TN, United States; Department of Emergency Medicine, Washington University School of Medicine, Saint Louis, MO, United States; Department of Host-Microbe Interactions, St. Jude Children’s Research Hospital, Memphis, TN, United States; Department of Pathobiological Sciences, University of Wisconsin–Madison, Madison, WI, United States; Department of Pathology and Immunology, Washington University School of Medicine, Saint Louis, MO, United States; Department of Bone Marrow Transplant and Cellular Therapy, St. Jude Children’s Research Hospital, Memphis, TN, United States; Department of Host-Microbe Interactions, St. Jude Children’s Research Hospital, Memphis, TN, United States; Division of Pulmonary and Critical Care Medicine, Department of Medicine, Washington University School of Medicine, Saint Louis, MO, United States; Department of Pathology and Immunology, Washington University School of Medicine, Saint Louis, MO, United States; Department of Pathology and Immunology, Washington University School of Medicine, Saint Louis, MO, United States; Department of Pathobiological Sciences, University of Wisconsin–Madison, Madison, WI, United States; Division of Pulmonary and Critical Care Medicine, Department of Medicine, Washington University School of Medicine, Saint Louis, MO, United States; Department of Pathology and Immunology, Washington University School of Medicine, Saint Louis, MO, United States; The Andrew M. and Jane M. Bursky Center for Human Immunology and Immunotherapy Programs, Washington University School of Medicine, Saint Louis, MO, United States; Center for Vaccines and Immunity to Microbial Pathogens, Washington University School of Medicine, Saint Louis, MO, United States; Department of Host-Microbe Interactions, St. Jude Children’s Research Hospital, Memphis, TN, United States; Center for Infectious Diseases Research, St. Jude Children’s Research Hospital, Memphis, TN, United States; Department of Microbiology, Immunology and Biochemistry, University of Tennessee Health Science Center, Memphis, TN, United States; Department of Emergency Medicine, Washington University School of Medicine, Saint Louis, MO, United States; The Andrew M. and Jane M. Bursky Center for Human Immunology and Immunotherapy Programs, Washington University School of Medicine, Saint Louis, MO, United States; Center for Vaccines and Immunity to Microbial Pathogens, Washington University School of Medicine, Saint Louis, MO, United States

**Keywords:** human, lung, T-cell receptors, T cells, viral infections

## Abstract

Lung tissue-resident CD8^+^ T cells facilitate viral clearance and protective immunity to influenza viruses in animal models. Their role during acute human infection is not clear. Here we use bronchoalveolar lavage samples collected from human subjects naturally infected with influenza B virus to show that influenza-specific CD8^+^ T cells are expanded in the lower airways during acute infection and target only a few epitopes from phylogenetically conserved internal influenza virus proteins. The lower airway influenza-specific CD8^+^ T-cell immunodominance hierarchy is different from the hierarchy observed in matched blood samples. Transcriptional and protein-level analyses using HLA class I tetramers reveal a tissue-resident profile and less expression of cytotoxic effector molecules in lower airway influenza-specific CD8^+^ T cells. Collectively, our data show that high-frequency influenza-specific CD8^+^ T cells with a tissue-resident phenotype are found in the lower airways during viral clearance. These cells recognize a handful of conserved viral epitopes and exhibit a functional phenotype different from cells found in blood. These cells may play a role in controlling human influenza infection.

## Introduction

Mucosal tissue-resident T cells represent a key barrier of the immune system that can prevent severe disease following breach of upstream defenses.^1^^,^^2^ Their importance has been well established in animal models of respiratory viral infection where they mediate cross-protection against variant pathogens in the absence of effective antibody responses^3^^,^^4^ and can even prevent respiratory virus transmission.^5^ The molecular phenotype and role of antigen-specific mucosal tissue-resident T cells in protection from human disease is less clear and has been difficult to study due to sampling limitations during acute infection.

HLA class I tetramers have been used in 2 previous clinical studies evaluating responses in the lower airways of influenza-infected humans.^6^^,^^7^ Those investigators discovered high-magnitude, influenza-specific T-cell responses in the small number of total cells present in bronchoalveolar lavage fluid (BALF) of 2 patients with severe naturally acquired seasonal influenza illness^6^ and in 5 subjects with mild experimental influenza infection.^7^ In addition, high-magnitude influenza-specific mucosal tissue-resident T cells have been characterized using HLA class I tetramers in uninfected lung tissue from deceased donors.^8^^,^^9^ Unfortunately, HLA class I tetramers do not allow measurement of the breadth and magnitude of all major antigen-specific CD8^+^ T-cell populations in a tissue. HLA class I tetramers also do not allow complete evaluation of the phenotype of human lung tissue-resident T cells. In the present work, we sought to agnostically sample the entire lower airway T-cell repertoire following naturally acquired seasonal influenza infection using single-cell RNA sequencing (scRNA-seq) with paired T-cell receptor (TCR) sequencing to determine the magnitude, diversity, epitope specificity, and phenotype of human influenza-specific T cells in the bronchoalveolar space.

## Materials and methods

### Human subjects

Human subjects were enrolled during the 2019–2020 influenza season. Demographics of the larger clinical cohort have been previously described.^10^ All 4 subjects exhibited symptoms at the time of study enrollment and tested positive for influenza B virus (IBV) on a clinical influenza test. Research blood samples and nasopharyngeal (NP) swab samples were obtained from the outpatient subjects at study enrollment. Following study enrollment, BALF samples from the 3 subjects with outpatient illness were obtained during a scheduled elective outpatient research bronchoscopy procedure performed on the indicated day after self-reported onset of influenza illness symptoms.

For outpatient bronchoscopy, following written informed consent, safety screening with blood coagulation studies, and a screening chest X-ray, subjects received intravenous conscious sedation and lidocaine was applied to the upper airway and vocal cords to facilitate safe and comfortable bronchoscopy. A brief visual inspection of the airways was performed to select an appropriate location for BALF collection; however, all outpatient BALF samples in this study were collected from the right middle lobe bronchus. Outpatient BALF samples were collected by the instillation of 150 mL of sterile saline in 3 separate 50-mL aliquots, and all returned lavage fluid was pooled together. Matched research blood samples were obtained at the time of the outpatient bronchoscopy procedure.

We obtained written informed consent from the legally authorized representative for the subject with severe IBV infection to obtain portions of a BALF sample obtained for clinical purposes and a matching research blood sample. The severe subject was intubated and mechanically ventilated prior to arrival at the study institution’s intensive care unit after being diagnosed with respiratory failure and IBV infection at an outside hospital. A clinically indicated BALF sample was obtained by the severe infection subject’s clinical team to evaluate for bacterial coinfection. The clinical team performed a single 50-mL wash with sterile saline in the right middle lobe bronchus that was discarded followed by two 50-mL lavages of the same right middle lobe bronchus with sterile saline that were pooled. The study team received approximately 20 mL of BALF fluid from the pooled lavage sample and the remainder was sent to the clinical laboratory. A matched blood sample was obtained from the severe subject’s intravenous catheter for research at the time of the bronchoscopy procedure.

### Sample preparation for scRNA-seq

Peripheral blood mononuclear cells (PBMCs) were prepared from blood samples obtained in EDTA-anticoagulated collection tubes by density gradient centrifugation. Contaminating red blood cells were removed by hypotonic lysis. After washing, PBMCs were counted and cryogenically preserved.

BALF samples were centrifuged at 400 × *g* for 15 minutes at 4 °C. BALF cellular material was resuspended in 4 mL of 40% Percoll and layered into a Percoll gradient column: 2 mL of 100% Percoll was placed into the bottom of a 15 mL sterile conical centrifuge tube, 2 mL of 60% Percoll was layered over that, the resuspended cells in 4 mL of 40% Percoll were layered next, and finally 2 mL of 30% Percoll was layered on top. The column was spun at 2000 × *g* for 20 minutes at room temperature and the immune cells were visualized and obtained from the interface between the 40% and 60% Percoll layers. Percoll-purified BALF immune cells were washed and cryogenically preserved.

Non-naïve CD8^+^ T cells were FACS sorted from cryopreserved PBMCs using a FACSAriaII (BD Biosciences) cell sorter. The following antibodies were employed: CD3 FITC (clone HIT3a, BioLegend), CD8 PE (clone RPA-T8, BioLegend), CD45RO Pacific Blue (clone UCHL1, BioLegend), CCR7 APC-R700 (clone 3D12, BD Biosciences), and live-dead discrimination with Zombie Aqua Fixable Viability Kit (BioLegend). We sorted all live CD3^+^CD8^+^ PBMCs that were not CD45RO^−^CCR7^+^ for inclusion in the scRNA-seq. Total cryopreserved Percoll-purified BALF immune cells were thawed and further purified using the EasySep Dead Cell Removal (Annexin V) Kit (STEMCELL Technologies) prior to scRNA-seq.

### Single-cell RNA sequencing

Following purification, non-naïve CD8^+^ T cells or BALF cells were resuspended in phosphate-buffered saline (PBS) supplemented with 0.05% bovine serum albumin. Chromium Single Cell 5′ v2 Gene Expression Dual Index libraries and Chromium Single Cell V(D)J Dual Index libraries (10x Genomics) were prepared according to the manufacturer’s instructions without modifications. Gene expression and V(D)J libraries were sequenced on a NovaSeq S4 flow cell (Illumina) using a NovaSeq 6000 instrument (Illumina), targeting a sequencing depth of 50,000 and 5000 read pairs per cell.

### TCR transductants

TRA and TRB V(D)J sequences of interest were combined in silico with mouse constant regions modified to include additional cysteine residues in place of serine at position 57 in mouse TRBC2 and threonine at position 47 in mouse TRAC to improve TRA/TRB association. Constructs were synthesized (GenScript) to contain TRA and TRB sequences separated by a T2A sequence with NotI and EcoRI restriction sites at the 5′ and 3′ ends of the region of interest. Synthesized constructs were double digested with NotI and EcoRI and cloned into the pMP71 retroviral vector; ligation was confirmed via sequencing of the recombinant plasmid. Recombinant pMP71 was used to transfect the 293Vec-RD114 retroviral packaging cell line (provided by BioVec Pharma) with TransIT-LT1 (Mirus Bio) transfection reagent using the manufacturer’s protocol and recommended conditions. Transfection medium was removed after 24 hours and replaced with fresh medium, and retrovirus-containing supernatants were collected 24 hours later. Retroviral supernatants were stored at −80 °C until use.

To generate primary human CD8^+^ T-cell transductants, we enriched cryopreserved PBMCs using an EasySep Human CD8 Positive Selection Kit II (STEMCELL Technologies). Isolated CD8^+^ T cells were cultured in R10-500 (R10 is composed of RPMI 1640 media supplemented with penicillin-streptomycin and 10% v/v heat-inactivated fetal bovine serum [FBS]; R10-500 is further supplemented with 500 U/mL recombinant human IL-2 [BioLegend]) at 37 °C with 5% CO_2_ and activated with the Miltenyi Biotec Human T Cell Activation/Expansion kit according to the manufacturer’s instructions. Two days after activation/expansion, activated CD8^+^ T cells were purified by density gradient centrifugation. Cells were washed in R10, resuspended at 2 × 10^6^ per mL in R10-500, and plated on 24-well flat-bottom tissue culture plates. TCR RD114 retroviral supernatants were thawed, layered on top of a 20% sucrose (w/v) cushion, and centrifuged in a microcentrifuge at 20,000 × *g* at 4 °C for 1 hour. The supernatant was discarded, and the residual volume, including the retroviral pellet, was incubated with ViroMag beads (OZ Biosciences) for 15 minutes at room temperature. Retrovirus/beads were then added to the activated T cells in a 24-well plate, and the plate was briefly centrifuged at 1600 × *g* for 1 minute before being placed on a prewarmed magnet (OZ Biosciences) and incubated at 37 °C with 5% CO_2_ for 15 minutes to facilitate transduction. Transduced CD8^+^ T cells were cultured for at least 1 week before analysis.

To generate TCR-transduced Jurkat reporter T cells, human Jurkat clone E6-1 T cells (ATCC) were transduced with an NFAT eGFP reporter lentivirus (BPS Bioscience), according to the manufacturer’s instructions. NFAT-GFP reporter Jurkat T cells were then transduced with a human CD8 alpha/beta RD114 retrovirus that we designed with a T2A site between the CD8 alpha and CD8 beta chains, had gene synthesized (GenScript), cloned the synthesized construct into the NotI/EcoRI sites of the pMP71 vector, and packaged in 293Vec-RD114 cells as described above for TCR retroviruses. Following sucrose purification and transduction using ViroMag beads, CD8^+^ NFAT-GFP reporter Jurkat T cells were sort purified on a Bigfoot spectral cell sorter (Invitrogen) using the antibodies CD3 PE-Cy7 (clone UCHT1, BioLegend) and CD8 BV421 (clone RPA-T8, BioLegend) and cultured under limiting dilution conditions, and the resulting CD8^+^ NFAT-GFP reporter Jurkat T-cell lines were confirmed to express human CD8 on the cell surface following culture by flow cytometry. Candidate TCRs were transduced into the CD8^+^ NFAT-GFP reporter Jurkat cells with ViroMag beads using the TCR retroviruses described above following sucrose purification with the exception that CD8^+^ NFAT-GFP reporter Jurkat cells were grown in R10 alone without IL-2 supplementation and CD8^+^ NFAT-GFP reporter Jurkat cells did not require activation prior to transduction. TCR-transduced CD8^+^ NFAT-GFP reporter Jurkat T-cell lines were purified using a PE-labeled murine TCR β-chain–specific monoclonal antibody (clone H57-597, BioLegend) and the EasySep PE Positive Selection kit II (STEMCELL Technologies) per manufacturer’s instructions. PE bead purified CD8^+^ NFAT-GFP reporter Jurkat T-cell lines were then cultured under limiting dilution conditions until pure clonal lines expressing the transduced TCR of interest were confirmed by flow cytometry using the murine TCR β-chain–specific monoclonal antibody and sequence-confirmed using genomic DNA from the resulting lines.

### Peptides

Peptides were provided by BEI Resources (National Institutes of Health) or purchased from GenScript. BEI resources provided overlapping peptides for B/Florida/4/2006 nucleoprotein (NR-36045, BEI Resources), B/Florida/4/2006 matrix protein 1 (NR-36046, BEI Resources), B/Brisbane/60/2008 neuraminidase protein (NR-19253, BEI Resources), and B/Brisbane/60/2008 hemagglutinin (HA) protein (NR-19247, BEI Resources). GenScript’s peptide library service synthesized 15-mer peptides overlapping by 11 amino acids covering the entire amino acid sequence of B/Brisbane/60/2008 PB1, PB2, PA, BM2, and NS1 proteins to ≥70% purity. All peptides were provided lyophilized and were solubilized at a stock concentration of 2 mg/mL in 10% DMSO and 90% sterile PBS. Megapools of up to 100 peptides and scanning pools of 10 peptides were generated for IBV response mapping.

### Reverse epitope mapping of TCR transductants

To reverse epitope map primary human CD8^+^ T-cell TCR transductants, 2.5 × 10^5^ to 5 × 10^5^ transduced T cells were cocultured with 1 × 10^5^ Epstein–Barr virus–transformed B lymphoblastoid cells (BLCLs) from the subject who expressed the index paired TCR in the presence of various mapping pools of IBV overlapping peptides. Each peptide was incubated at a final concentration of 1 µg/mL. Separate unstimulated control wells with equivalent concentrations of DMSO to the final concentration of DMSO found in the peptide-stimulated condition were included. Positive control phorbol 12-myristate 13-acetate (InvivoGen) and ionomycin (InvivoGen) were added to a separate well. Cells in all conditions were cocultured in R10 medium supplemented with co-stimulatory antibodies to CD28 and CD49d (BD Biosciences). Samples were incubated for 1.5 hours before the addition of brefeldin A and monensin (both from BD Biosciences) and incubated for an additional 12 to 16 hours. Surface staining was performed, followed by fixation in 1% paraformaldehyde for 15 minutes, permeabilization with FACS washing buffer (PBS pH 7.4 without magnesium or calcium [Gibco], at a final concentration of 2% heat-inactivated FBS v/v and 2 mM EDTA) supplemented with 0.1% (w/v) saponin (Sigma) and intracellularly stained with fluorescently labeled antibodies directed to cytokine antigens. We used the following antibodies: CD3 PE-Cy7 (clone UCHT1, BioLegend), CD8 BUV 563 (clone RPA-T8, BD Biosciences), mouse TCR β-chain APC-Cy7 (clone H57-597, BioLegend), CD69 BV711 (clone FN50, BioLegend), IFN-γ PE (clone B27, BioLegend), TNF-α PerCP-Cy5.5 (clone MAb11, BioLegend), and IL-2 APC (clone 5344.111, BD Biosciences). The panel included Zombie NIR viability stain (BioLegend). All antibodies were used at pretitrated optimal staining concentrations. Cells were run on a 5-laser Cytek Aurora spectral flow cytometer. Following spectral unmixing on the instrument in SpectroFlo software (version 3.3.0, Cytek), unmixed .fcs files were analyzed using FlowJo software (version 10, BD Biosciences).

To reverse epitope map NFAT-GFP reporter CD8^+^ Jurkat T-cell line TCR transductants, 2.5 × 10^5^ to 5 × 10^5^ reporter Jurkat cells expressing the TCR of interest were cocultured in R10 with 1 × 10^5^ BLCLs from the subject who expressed the index paired TCR of interest in the presence or absence of various mapping pools of IBV overlapping peptides at a final concentration of 1 µg/mL for each peptide for a total of 16 hours at 37 °C and 5% CO_2_. Following coculture, cells were examined for GFP expression on a 5 laser Cytek Aurora spectral flow cytometer. GFP expression was analyzed using FlowJo software (version 10, BD Biosciences).

### HLA restriction determination

We determined HLA restriction for reverse epitope–mapped TCR responses using single HLA class I allele–expressing K562 cells. To generate novel HLA class I allele–expressing K562 cells for the included subjects, we synthesized each required class I allele along with the human beta-2-microglobulin gene separated by a T2A site (GenScript). Synthesized constructs were cloned into a pMP71 retroviral transduction plasmid as described above and transfected into the 293Vec-RD114 retroviral packaging cell line. Resulting retroviruses were used to transduce K562 cells (ATCC), which are devoid of surface-expressed HLA-A and HLA-B under normal culture conditions and express very low amounts of endogenous surface HLA-C. Single HLA allele–transduced K562 cells were purified using an anti-human HLA class I antibody labeled with PE (clone W6/32, BioLegend) and the EasySep PE Positive Selection kit II (STEMCELL Technologies) per manufacturer’s instructions. PE bead–purified single HLA class I allele–expressing K562 cell lines were then cultured under limiting dilution conditions until pure clonal lines expressing the transduced HLA allele of interest were confirmed by flow cytometry using the anti-human HLA class I–specific monoclonal antibody (clone W6/32, BioLegend) and sequencing of genomic DNA from the resulting lines with HLA retroviral insert-specific primers.

To determine HLA restriction, 2.5 × 10^5^ to 5 × 10^5^ TCR-transduced primary human CD8^+^ T cells or NFAT-GFP reporter Jurkat TCR line cells were cocultured in R10 with 1 × 10^5^ K562 cells expressing the individual HLA-A, -B, or -C alleles expressed by the subject from whom the TCR of interest was obtained, or non-HLA-transduced K562 cells in the presence or absence of 10 µg/mL peptide of the previously mapped TCR response using the culture and FACS staining conditions described above. Notably, high background was obtained on TCR with epitopes restricted by HLA-A*03:01—an allele endogenously expressed by the Jurkat cell line—and some HLA-C allele restricted epitopes whose HLA share substantial amino acid sequence similarity to the endogenous K562 HLA-C alleles (specifically HLA-C*03:07).

### HLA class I tetramer analysis

HLA class I monomers loaded with the indicated peptides were synthesized by the Bursky Center for Human Immunology and Immunotherapy Immunomonitoring Lab core facility at Washington University in Saint Louis. Peptide-loaded and biotinylated HLA class I monomers were tetramerized by gradual addition of calculated equimolar amounts of streptavidin–R-PE (PJRS27-1, Agilent Technologies). Tetramerized peptide-loaded and control unloaded PE-labeled HLA class I tetramer reagents were tested and titrated on untransduced CD8^+^ Jurkat T-cell lines and TCR-transduced CD8^+^ Jurkat T-cell lines expressing the TCR recognizing the epitope of interest.

For HLA tetramer intracellular staining, >1 million PBMCs and all remaining BALF cellular material that had been cryopreserved from the indicated subjects were stained first with titrated HLA class I tetramer for 15 minutes at room temperature. Then, without washing the tetramer away, previously titrated surface staining antibodies were added together as a master mix contained in BD Horizon Brilliant Stain Buffer (BD Biosciences): CD3 BUV 805 (clone UCHT1, BD Biosciences), CD8 Spark Plus UV 395 (clone RPA-T8, BioLegend), CD45RO Pacific Blue (clone UCHL1, BioLegend), CCR7 Brilliant Violet 785 (clone G043H7, BioLegend), CD69 BV605 (clone FN50, BioLegend), and CD103 BV510 (clone Ber-ACT8, BD Biosciences). Zombie NIR fixable viability stain (BioLegend) was included. Surface stains were incubated with the samples on ice for 30 minutes prior to washing, fixing, and permeabilization with Foxp3/Transcription Factor Staining Buffer Set (Invitrogen) according to the provided protocol. Intracellular antibodies were then added: IFN-γ BV650 (clone 4S.B3, BioLegend), TNF-α PerCP-Cy5.5 (clone Mab11, BioLegend), perforin cFluor R720 (clone dG9, Cytek), granzyme B APC-Cy7 (clone QA16A02, BioLegend), and granzyme K FITC (clone GM26E7, BioLegend). Samples were run on a 5 laser Cytek Aurora spectral flow cytometer. Following spectral unmixing on the instrument in SpectroFlo software (version 3.3.0, Cytek), unmixed .fcs files were analyzed using FlowJo software (version 10, BD Biosciences).

### Autologous virus sequencing

Viral RNA was extracted from 300 µL of NP swab universal transport media or BALF using the Viral Total Nucleic Acid Purification kit (Promega) on a Maxwell RSC 48 instrument and eluted in 50 µL of nuclease-free water according to the manufacturer’s instructions. For RT-PCR, 3 µL of viral RNA was added to a 25-µL reaction using the SuperScript III HiFi Enzyme Mix (Invitrogen) 0.5 µL, 12.5 µL 2× reaction mix, 7 µL nuclease-free water, and 2 µL of 10 µM IBV-GA2 universal primer pool targeting the conserved ends of the IBV gene segments. The IBV-GA2 pool contained the following primers: B-PBs-UniF—GGG GGG AGC AGA AGC GGA GC (relative molar ratio 1.5), B-PBs-UniR—CCG GGT TAT TAG TAG AAA CAC GAG C (relative molar ratio 1.5), B-PA-UniF—GGG GGG AGC AGA AGC GGT GC (relative molar ratio 1), B-PA-UniR—CCG GGT TAT TAG TAG AAA CAC GTG C (relative molar ratio 1), B-HANA-UniF—GGG GGG AGC AGA AGC AGA GC (relative molar ratio 1.2), B-HANA-UniR—CCG GGT TAT TAG TAG TAA CAA GAG C (relative molar ratio 1.2), B-NP-UniF—GGG GGG AGC AGA AGC ACA GC (relative molar ratio 1.5), B-NP-UniR—CCG GGT TAT TAG TAG AAA CAA CAG C (relative molar ratio 1.5), B-M-Uni3F—GGG GGG AGC AGA AGC ACG CAC TT (relative molar ratio 0.6), B-Mg-Uni3F—GGG GGG AGC AGA AGC AGG CAC TT (relative molar ratio 0.6), B-M-Uni3R—CCG GGT TAT TAG TAG AAA CAA CGC ACT T (relative molar ratio 1.2), B-NS-Uni3F—GGG GGG AGC AGA AGC AGA GGA TT (relative molar ratio 1.5), and B-NS-Uni3R—CCG GGT TAT TAG TAG TAA CAA GAG GAT T (relative molar ratio 1.5). Temperature cycle conditions were as follows: 42 °C for 50 minutes; 50 °C for 10 minutes; 94 °C for 2 minutes; 4 cycles of 94 °C for 30 seconds, 43 °C for 30 seconds, and 68 °C for 3 minutes 50 seconds; and 30 cycles of 94 °C for 30 seconds, 57 °C for 30 seconds, and 68 °C for 3 minutes 30 seconds (extending this last step by 10 seconds per cycle). DNA was purified with 1× Ampure beads. DNA concentrations were determined using a Qubit dsDNA High Sensitivity Assay Kit on a Qubit Fluorometer (Invitrogen). One nanogram of DNA was used as input for the Nextera DNA Library Prep kit, which was used to tagment samples according to the manufacturer’s instructions. Tagmented and amplified products were purified with the AMPure XP paramagnetic beads for PCR Purification kit (Beckman Coulter) in 2 consecutive steps (0.5× and 0.7×) and were quantified using the Qubit dsDNA high-sensitivity kit (Invitrogen). Sample quality control was performed using the Bioanalyzer High Sensitivity DNA Analysis Kit on a Agilent 2100 Bioanalyzer (Agilent). All samples were prepared and sequenced in technical replicate using the same RNA.

Generated libraries were sequenced using 2 × 150 bp paired-end reads on an Illumina NovaSeq, generating at least 2 million paired-end reads for each sample. Before proceeding with analysis, we ensured that <1× coverage of the IBV genome was generated in negative controls. Raw sequencing files are available at Sequence Read Archive BioProject PRJNA1240834 (biosamples SAMN47541152, SAMN47541153, SAMN47541154, and SAMN47541155). Consensus sequences are available under GenBank accession numbers PV382145–PV382152.

### Autologous virus analysis

Intrahost single-nucleotide variants (iSNVs) were called using bespoke scripts. All read processing scripts were executed through the rieshunter/tcflab: v2.04 Docker image (https://hub.docker.com/r/rieshunter/tcflab/tags). This Docker image consists of a Linux GNU operating system (18.04.6 LTS “Bionic Beaver”) with all necessary commands and program files (Docker version 20.10.13).

Raw, paired FASTQ files from samples positive for the subtype of interest were adapter-trimmed and quality-trimmed to an average 5-base sliding-window quality of Q20 using Trimmomatic (version 0.39). Additionally, all reads <100 bases in length were discarded. Trimmed, paired reads were then merged using BBMerge.sh (BBMap version 38.96), outputting merged pairs and unmerged files. Merged and unmerged reads were normalized to 2,000 reads for all 31-mers above 200 depth. Normalized, merged, and unmerged reads were aligned to the reference sequence, using bwa mem (BWA version 0.7.17). Alignments were concatenated with samtools merge to produce a single sample alignment file from the merged file and the R1 and R2 unmerged files (Samtools version 1.15). iSNVs were called using callvariants.sh (BBMap version 38.96) to a minimum Phred quality score of Q30 and minimum position coverage of 100. A minimum frequency of 3% was applied after post-processing analysis in RStudio. Consensus sequences were generated by applying variants above 50% to the reference sequence using BCFtools (version 1.15). The vaccine strain for the 2019–2020 flu season, B/Iowa/06/2017/Victoria, was used as the reference sequence. Consensus sequences from sample alignments that did not map to one or more gene segments, did not have ≥100× coverage at one or more segments, or did not have ≥99% coverage of the complete genome were discarded. Variants were annotated using snpEff (version 5.1) relative to the reference sequence to categorize mutation type (synonymous, nonsynonymous, stop-gained, etc.).

VCF files from the Nextflow pipeline were cleaned using a custom R script (R version 4.4.2). Variants were filtered to include only nonsynonymous and synonymous variants. iSNVs were required to be at a frequency of 3% and have a depth of coverage of at least 200 reads. iSNVs were required to be present in both technical replicates. iSNVs were compared to the reference strain B/Iowa/06/2017/Victoria.

### TCR sequence analysis and visualization

Paired TCR sequences obtained for each BALF or blood sample from the Cell Ranger output TCR clonotype files were parsed and merged on identical paired TCRs from each of the 2 separate runs performed for each BALF sample using the data.table package in R (version 4.3.3). Treemap plots were created using the ggplot2 and treemapify libraries in R.

### HLA typing

HLA typing was performed using the AllType NGS 11-Loci Amplification Kit (One Lambda Lab Solutions, lot #013) according to manufacturer’s instructions. Resulting libraries were sequenced on a MiSeq lane at 150 × 150 bp (Illumina). HLA types were called using TypeStream Visual software (v2.0.1; One Lamba).

### scRNA-seq analysis

Single-cell data were parsed with CellRanger (v6.1.2, 10x Genomics) using the GRCh38-2020-A reference for gene expression sequencing and with CellRanger (v6.0.1) using the GRCh38-alts-ensembl-5.0.0 reference for VDJ sequencing. Gene expression data were inspected for quality at this stage (mean reads per cell, median genes per cell, fraction of reads in cells) and then aggregated via CellRanger *aggr* using mapped read normalization. Downstream analysis was done separately in clonotype neighbor graph analysis (CoNGA)^11^ and Seurat^12^ (v5.1.0). TCR data were incorporated into the Seurat analyses using scRepertoire^13^ (v2.0.5). Standard single-cell processing followed, as has been described elsewhere.^14^ To exclude dead or dying cells, we filtered out cells with ≥15 percent of expression owed to mitochondrial genes or <300 genes detected. To exclude putative doublets, we filtered cells from each reaction that were above the 98th percentile for number of RNA molecules detected or number of genes detected for that reaction. Cell cycle scores were generated using markers identified previously.^15^ Data were log normalized, and variable features were detected using the “vst” method after excluding for genes associated with VDJ segments and scaled to regress out the effects of cell cycle scores, number of RNA molecules detected, and mitochondrial expression. We used PCA and UMAP dimensionality reduction for visualization and default approaches in Seurat to infer transcriptional clusters. Cells from lung samples were annotated using SCType,^16^ but downstream analyses were conducted on CD8^+^ T cells as annotated by canonical markers CD3E and CD8A and the detection of a paired αβ TCR. Differential gene expression was tested using the FindMarkers() function in Seurat. Gene set enrichment analysis was conducted using the *escape* package.^17^

### Study approval

The Washington University in Saint Louis School of Medicine Institutional Review Board (IRB) reviewed and approved the clinical study (IRB approval numbers 201808115 and 201910011). Written informed consent was obtained from each research participant or their legally authorized representative.

## Results

### Clinical cohort

We completed a prospective observational clinical study of influenza-infected adult subjects. We collected matched blood and BALF from most enrolled subjects. The clinical cohort, including information about the cellular composition and cytokine concentrations of BALF, has been previously described.^10^ Using available samples from the larger clinical cohort, we selected 3 subjects who experienced symptomatic, but relatively mild, IBV infection and a fourth age- and sex- matched subject with severe IBV infection requiring 10 days of invasive mechanical ventilation in the intensive care unit (Table 1) for further study using scRNA-seq techniques.

**Table 1:** Human subject demographics and HLA typing.

Subject ID	Age, y	Sex	Days after symptom onset for blood/BALF collection	Medical comorbidities	Severity	HLA-A	HLA-B	HLA-C
**1920B004**	29	F	13	None	Outpatient	01:01/74:01	40:01/50:01	03:04/06:02
**1920B007**	28	F	20	None	Outpatient	01:01/02:01	08:01/44:02	03:04/07:01
**1920B013**	41	M	14	None	Outpatient	02:01/23:01	18:01/44:02	04:01/07:04
**1920B011**	26	F	7	Asthma, diabetes, obesity	ICU, intubated, survived	02:01/03:01	14:02/35:12	04:01/08:02

### Paired TCR sequencing and discovery of IBV-specific CD8^+^ T cells

**Figure 1:**
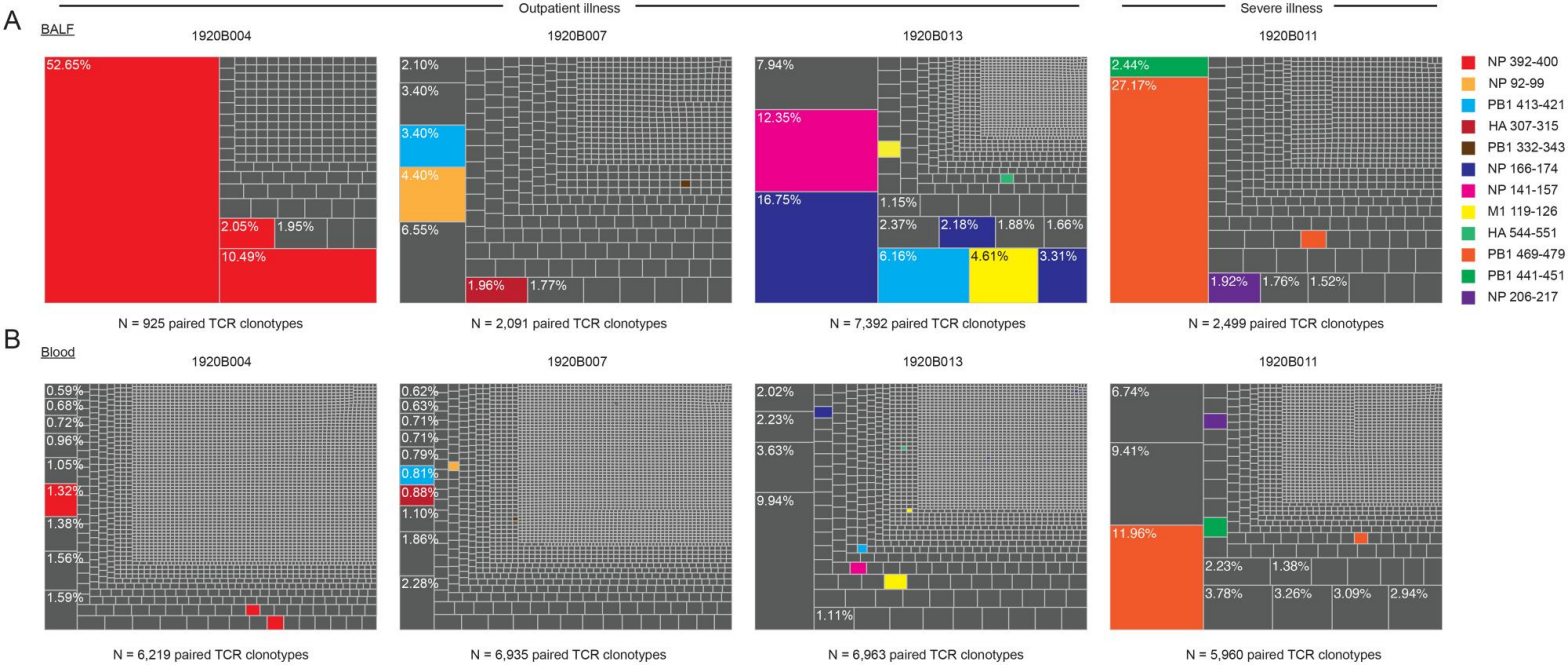
Focused clonally expanded lower airway paired TCR repertoire following IBV infection. The size of each box represents the proportion of that paired TCR among all unique paired TCRs sequenced. Reverse epitope–mapped IBV-specific paired clonotypes are denoted by colors. (A) Paired clonotypes found in unsorted BALF T cells. (B) Paired clonotypes found in temporally matched PBMC samples FACS sorted on all CD8^+^ T cells that are not CD45RO^−^CCR7^+^ (naïve) prior to single-cell sequencing. HA, hemagglutinin; M1, matrix protein 1; NP, nucleoprotein; PB1, polymerase basic 1.

We performed scRNA-seq and paired TCR sequencing on temporally matched samples of total BALF cellular material and FACS-sorted non-naïve CD8^+^ T cells from the blood of these 4 subjects. In each of the 4 subjects, paired TCR sequencing of BALF T cells revealed between 2 and 8 major clonal TCR expansions encompassing 2% or more of total paired TCRs (Fig. 1A). The largest clonal TCR expansions encompassed between 7% and 53% of all paired BALF TCR sequences (Fig. 1A). Many of these clonally expanded BALF TCRs were detected at much lower frequency in blood non-naïve CD8^+^ T cells (Fig. 1B).

To determine the antigen specificity of these clonally expanded T-cell populations, we synthesized 18 paired TCR clonotypes found at a frequency of 2% or higher in the BALF that were also found in the non-naïve CD8^+^ T-cell blood population and 5 paired TCRs sharing characteristics of antigen-specific CD8^+^ T cells found in both BALF and blood datasets using the CoNGA algorithm^11^ (Table 2). Additionally, we synthesized a paired TCR clonotype (TCR 24) found in 10% of sorted non-naïve CD8^+^ T cells from blood in subject 1920B013 that was detected at low frequency in matched BALF (Fig. 1B and Table 2). Reverse epitope mapping of these TCRs in NFAT-GFP reporter Jurkat T cells (Fig. S1) and primary human CD8^+^ T cells (Fig. S2) using sets of overlapping IBV 15- to 17-mer peptides overlapping by 11 or 12 amino acids revealed that the majority (19 of 24) were IBV-specific. Strikingly, almost all (17 of 19) IBV-specific TCRs recognized IBV epitopes found in phylogenetically conserved,^18^^,^^19^ internal IBV proteins (Fig. 1 and Table 3). Two of the 12 epitopes had been previously reported^19^; however, 10 of the discovered epitopes, including the highest frequency response in each subject, were novel and had not been previously reported in the Immune Epitope Database. Further analysis revealed that all 12 reverse epitope–mapped IBV epitopes, including 2 separate HA epitopes, were conserved in contemporarily circulating strains and in the ancestral B/Lee/1940 strain (Table 4). At least 4 of the 12 targeted lower airway epitopes also appeared conserved in ancestral H1N1, H2N2, and H3N2 influenza A viruses (Table 4), suggesting that high-frequency CD8^+^ T-cell responses in the lower airways may be selected in part by their ability to bind broadly cross-reactive epitopes found in multiple influenza strains. Nucleoprotein was the principal target of clonally expanded IBV-mapped CD8^+^ T cells in human BALF followed by the polymerase basic 1 (PB1) subunit of influenza polymerase (Fig. 1A and Table 3).

**Table 2:** Bronchoalveolar lavage CD8^+^ T-cell clonotypes selected for reverse epitope mapping.

Subject ID	TCR ID	TRAV/TRAJ	TRA CDR3	TRBV/TRBD/TRBJ	TRB CDR3	BALF (% of total paired TCR)	Blood (% of non-naïve CD8^+^ T cells)
**1920B011**	TCR 1	V24/J47	CAFRVDGNKLVF	V5-6/J1-6	CASSLAGSPLHF	27.171%	11.963%
	TCR 2^a^	V19/J20	CALSGPNDYKLSF	V28/J1-5	CASSLQGSNQPQHF	0.560%	0.252%
	TCR 3	V17/J11	CATDKGYSTLTF	V28/J1-1	CASKRQNTEAFF	1.921%	0.453%
	TCR 4	V13-1/J27	CAATRGAGKSTF	V27/J2-3	CASSFGTDTQYF	2.441%	0.570%
**1920B004**	TCR 5	V6/J39	CALGNHMLTF	V30/J1-1	CAWKTGGTEAFF	52.649%	1.329%
	TCR 6	V8-2/J9	CVVRVNTGGFKTIF	V28/J2-2	CASSFPERNTGELFF	10.486%	0.193%
	TCR 7	V14/J6	CAMRGAPSGGSYIPTF	V29-1/D1/J1-2	CSVGSVGLPDVSKGGYTF	2.054%	0.289%
**1920B013**	TCR 8	V26-1/J22	CIVRPGSARQLTF	V4-1/J1-5	CASSQDVQGWPQHF	16.748%	0.259%
	TCR 9	V21/J36	CAVHGGANNLFF	V20-1/J2-7	CSAGDLTYEQYF	12.351%	0.244%
	TCR 10	V13-2/J13	CAESSGGYQKVTF	V27/J2-7	CASSPGSSYEQYF	7.941%	0.029%
	TCR 11^a^	V22/J47	CAVPYGNKLVF	V4-1/J1-2	CASSQGAGENYGYTF	0.446%	0.029%
	TCR 12^a^	V29/J41	CAALSNSGYALNF	V4-3/J2-7	CASSQGQGRYEQYF	0.149%	0.029%
	TCR 13	V20/J39	CAVQAYAGNMLTF	V4-1/D1/J1-1	CASSLFGEGATEAFF	6.155%	0.100%
	TCR 14	V22/J43	CAPKPNNDMRF	V4-1/J2-7	CASSQGAGNHYEQYF	4.613%	0.416%
	TCR 15	V1-1/J17	CAVLQKAAGNKLTF	V27/J2-7	CASSPNEGSTYEQYF	3.314%	0.014%
	TCR 16	V8-3/J32	CAVGLNYGGATNKLIF	V13/J2-5	CASSLGLAGLEGTQYF	2.178%	0.014%
	TCR 24	V30/J52	CGTESPFAGGTSYGKLTF	V10-3/J2-2	CAIRDRVTSETTGELFF	0.027%	9.938%
**1920B007**	TCR 17	V16/J26	CALSPQAADNYGQNFVF	V5-1/D1/J1-1	CASSAGDRGVGTEAFF	6.552%	0.101%
	TCR 18	V38-2/J40	CAYRSGPASGTYKYIF	V27/D2/J2-1	CASSFSRTSGGGHEQFF	4.400%	0.115%
	TCR 19	V12-3/J10	CAIEGGGNKLTF	V12-4/J1-1	CASSLAGGLTEAFF	3.396%	0.807%
	TCR 20	V26-1/J54	CIVRVAASIVVQGAQKLVF	V20-1/D1/J1-3	CSAVPRDRGQGNTIYF	2.104%	0.058%
	TCR 21	V21/J31	CAAPTNARLMF	V5-6/J1-1	CASSSGGNTEAFF	1.961%	0.880%
	TCR 22^a^	V21/J50	CAVKTSYDKVIF	V6-5/J1-2	CASSYSGSGYTF	0.526%	0.144%
	TCR 23^a^	V21/J5	CAVMDTGRRALTF	V6-5/J1-1	CASSYSKFGGLNTEAFF	0.096%	0.029%

a TCR identified as putative antigen-specific TCR in both BALF and blood datasets using the CoNGA algorithm.^11^

**Table 3:** Bronchoalveolar lavage CD8^+^ T-cell epitopes.

Subject ID	TCR ID	HLA restriction	Specificity	Mapped influenza B amino acid sequence
**1920B011**	TCR 1^a^	A*03:01	PB1 469-479	CKLLGVNMSKK
	TCR 2^b^	A*03:01	PB1 470-479	KLLGVNMSKK^c^
	TCR 3^a^^,^^b^	B*14:02	NP 206-217	DVCFQRSKALKR
	TCR 4^b^	B*35:12	PB1 441-451	QSSDDFALFVN
**1920B004**	TCR 5^a^^,^^b^	C*06:02	NP 392-400	AAYEDLRVL^c^
	TCR 6^b^	C*06:02	NP 392-400	AAYEDLRVL^c^
	TCR 7^b^	C*06:02	NP 392-400	AAYEDLRVL^c^
**1920B013**	TCR 8^b^	A*23:01	NP 166-174	FSPIRITFL^c^
	TCR 9^a^	B*44:02	NP 141-157	GKEEIDHNKTGGTFYKM
	TCR 10	…	Unknown^d^	…
	TCR 11^b^	B*18:01	M1 119-126	HESSALLY^c^
	TCR 12^b^	A*02:01	HA 544-551	LDNHTILL^c^
	TCR 13^a^	A*02:01	PB1 413-424	FNMLSTVLGVAA
	TCR 14^b^	B*18:01	M1 119-126	HESSALLY^c^
	TCR 15^b^	A*23:01	NP 166-174	FSPIRITFL^c^
	TCR 16^b^	A*23:01	NP 166-174	FSPIRITFL^c^
	TCR 24	…	Unknown^d^	…
**1920B007**	TCR 17	…	Unknown^d^	…
	TCR 18^b^	B*08:01	NP 92-99	QMMVKAGL^c^
	TCR 19^b^	A*02:01	PB1 413-421	FNMLSTVLG^c^
	TCR 20	…	Unknown^d^	…
	TCR 21^b^	A*01:01	HA 307-315	EADCLHEKY^c^
	TCR 22	…	Unknown^d^	…
	TCR 23^b^	C*03:04	PB1 332-343	WFRDFCSIAPVL

Abbreviations: HA, hemagglutinin; M1, matrix protein 1; NP, nucleoprotein; PB1, polymerase basic 1.

a Reverse epitope mapped using primary cell line.

b Reverse epitope mapped using NFAT-GFP reporter CD8^+^ Jurkat T-cell line.

c Experimentally determined minimal epitope.

d Unknown TCRs were evaluated as TCR-transduced (1) NFAT-GFP reporter Jurkat CD8^+^ T-cell lines against pools of all overlapping IBV peptides, (2) primary cell lines for responsiveness to all overlapping IBV peptides, and (3) primary cell lines for responsiveness to IBV-infected autologous Epstein–Barr virus–transformed B-cell lines. Responses were not reliably or reproducibly detected above background in any of these 3 modalities, but we cannot discount the possibility that these TCRs may be IBV specific and our methods were just not sensitive enough to detect this specificity.

**Table 4:** Conservation of CD8^+^ T-cell epitopes across influenza virus strains.

Influenza virus strain	A*03:01 PB1_470-479_	B*14:02 NP_206-217_	B*35:12 PB1_441-451_	C*06:02 NP_392-400_
**B/Brisbane/60/2008 (B/Victoria)**	KLLGVNMSKK	DVCFQRSKALKR	QSSDDFALFVN	AAYEDLRVL
**B/Colorado/06/2017 (B/Victoria)**	−−−−I−−−−−	−−−−−−−−−−−−	−−−−−−−−−−−	−−−−−−−−−
**B/Minnesota/15/2020 (B/Yamagata)**	−−−−I−−−−−	−−−−−−−−−−−−	−−−−−−−−−−−	−−−−−−−−−
**B/Lee/1940**	−−−−I−−−−−	−−−−−−−−G−−−	−−−−−−−−−−−	−−−−−−−−−
**A/California/04/2009 (H1N1)**	−−V−I−−−−−	−ATY−−TR−−V−	−−−−−−−−I−−	−−F−−−−−S
**A/New York/392/2004 (H3N2)**	−−V−I−−−−−	−ATY−−TR−−V−	−−−−−−−−I−−	−−F−−−−L−
**A/Korea/426/1968 (H2N2)**	−−V−I−−−−−	−TTY−−TR−−V−	−−−−−−−−I−−	−−F−−−−−−

	A*23:01 NP_166-174_	B*44:02 NP_141-157_	B*18:01 M1_119-126_	A*02:01 HA_544-551_
**B/Brisbane/60/2008 (B/Victoria)**	FSPIRITFL	GKEEIDHNKTGGTFYKM	HESSALLY	LDNHTILL
**B/Colorado/06/2017 (B/Victoria)**	−−−−−−−−−	−−−−−−−−−−−−−−−−−	−−−−−−−−	−−−−−−−−
**B/Minnesota/15/2020 (B/Yamagata)**	−−−−−−−−−	−−−−−−−−−−−−−−−−−	−−−−−−−−	−−−−−−−−
**B/Lee/1940**	−−−−K−−−−	−−−−−−−−−−−−−−−−−	−−−−−−−−	−−−−−−−−
**A/California/04/2009 (H1N1)**	MRELX−LYD	−−XXX−PK−−−−PI−RR	YSTG−−AS	−ESTRIYQ
**A/New York/392/2004 (H3N2)**	MRELXVLYD	−−XXX−PK−−−−PI−RR	YSAG−−AS	YKDWILWI
**A/Korea/426/1968 (H2N2)**	MRELXVLYD	−−XXX−PK−−−−PI−−R	YSAG−−AS	MGVYQ−−A

	A*02:01 PB1_413-421_	B*08:01 NP_92-99_	A*01:01 HA_307-315_	C*03:04 PB1_332-343_
**B/Brisbane/60/2008 (B/Victoria)**	FNMLSTVLG	QMMVKAGL	EADCLHEKY	WFRDFCSIAPVL
**B/Colorado/06/2017 (B/Victoria)**	−−−−−−−−−	−−−−−−−−	−−−−−−−−−	−−−−−−−−−−−−
**B/Minnesota/15/2020 (B/Yamagata)**	−−−−−−−−−	−−−−−−−−	−−−−−−−E−	−−−−−−−−−−−−
**B/Lee/1940**	−−−−−−−−−	−−−−−−−−	−−−−−−−−−	−−−−−−−−−−−−
**A/California/04/2009 (H1N1)**	−−−−−−−−−	−−CTELK−	VH−−NTTCQ	−−−NIL−M−−IM
**A/New York/392/2004 (H3N2)**	−−−−−−−−−	−−CTELK−	KCNSECITP	−−−NIL−−−−IM
**A/Korea/426/1968 (H2N2)**	−−−−−−−−−	−−CTELK−	−TK−QTPLG	−−−NVL−−−−IM

### Validation of paired TCR sequencing results using HLA class I tetramers and FACS

**Figure 2:**
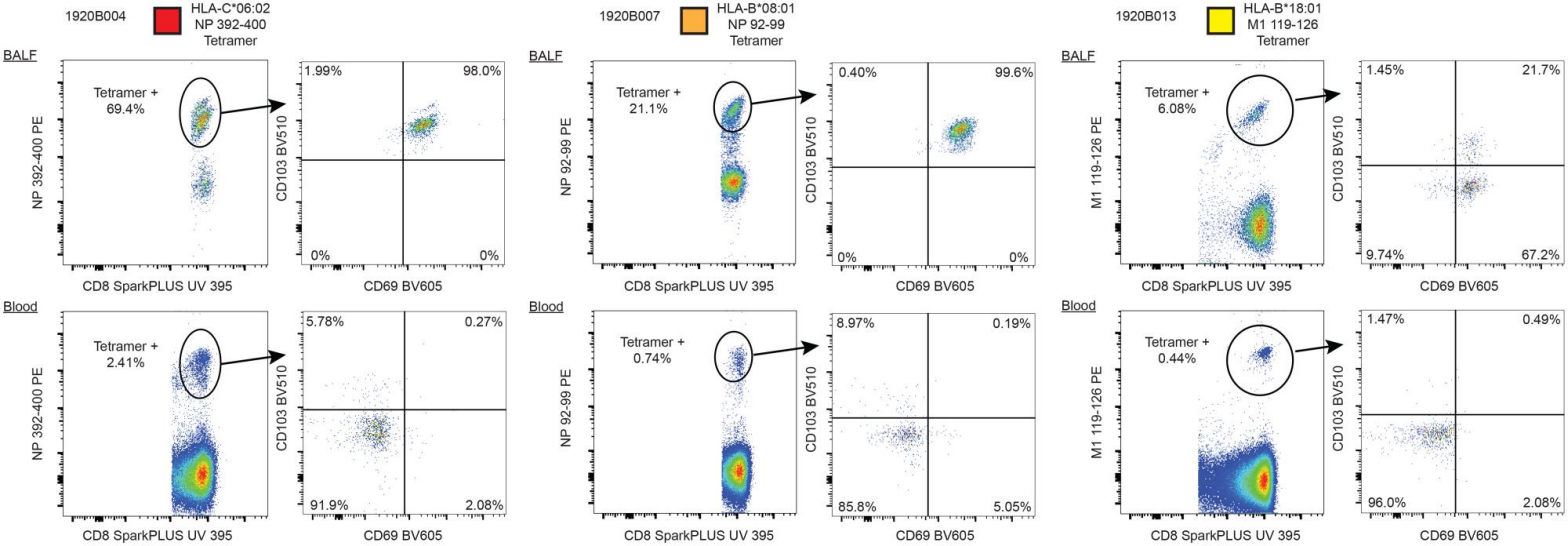
Flow cytometry of 3 outpatient subjects demonstrating the frequency of the indicated HLA class I tetramer-positive cells gated on total BALF CD3^+^CD8^+^ T cells (top) and CD3^+^CD8^+^ cells that are not CD45RO^−^CCR7^+^ (non-naïve CD8^+^ T cells, bottom). The frequency of tissue residency markers CD69 and CD103 are demonstrated on each gated tetramer-positive population.

To validate our reverse epitope mapping approach that agnostically characterized the immunodominance hierarchy of these BALF responses from the low number of available lymphocytes in the samples, we constructed HLA class I tetramers for novel immunodominant epitopes found at high frequency in the BALF and performed FACS analysis on the remaining cryopreserved cells from each available paired sample (Fig. 2). Using this approach, we confirmed that 69.4% of the total CD8^+^ T-cell population in the BALF of subject 1920B004 stained positive with the HLA-C*06:02 NP_392-400_ tetramer, which corresponded to the epitope-specific response shared by the 3 dominant TCR clonotypes discovered in the BALF of that subject, together composing >65% of all BALF TCR sequences in that sample (Fig. 2). Nearly all of these BALF tetramer-positive cells expressed the tissue residency markers CD69 and CD103 (Fig. 2).

The frequencies of HLA class I tetramer-positive CD8^+^ T cells in subjects 1920B004 and 1920B013 in both BALF and in blood non-naïve CD8^+^ T cells closely aligned with the frequencies of these populations that we discovered in our sequencing analysis (Fig. 1 and Table 2). The HLA-B*08:01 NP_92-99_ tetramer stained a much higher percentage of subject 1920B007’s BALF CD8^+^ T cells and blood non-naïve CD8^+^ T cells than predicted by the frequency of the single TCR sequence we characterized by reverse epitope mapping (Figs. 1 and 2 and Table 2). This finding suggests that multiple additional clonotypes responsive to this epitope were detected in the TCR sequencing in both the BALF and blood but were not reverse epitope mapped in our current analysis due to lower overall frequency as a percentage of total sequenced paired TCRs. The sequence for the TCR that binds NP_92-99_, TCR 18, was not closely related to any other BALF TCR sequences found in subject 1920B007 by TCRdist^20^ (data not shown). Therefore, the other clonotypes responsive to this epitope are likely structurally distinct from TCR 18. We also observed 3 very distinct TCR sequences for the TCR that bound the HLA-C*06:02 NP_392-400_ epitope in subject 1920B004 (TCR 5, TCR 6, and TCR 7; Table 2). Together, these observations may indicate that unique and structurally diverse epitope-specific clonotypic lineages undertake residence in the lower airways.

### Focused CD8^+^ T-cell response does not select for virus sequence variation

We next asked whether these strong, focused CD8^+^ T-cell responses we mapped in influenza-infected subjects could select for influenza viral sequence variation in the targeted CD8^+^ T-cell epitopes. We attempted to purify and sequence IBV viral RNA from available NP swab fluid obtained from each of the subjects. Viral RNA was not detectable by qPCR in a day 6 post–symptom onset NP swab sample from subject 1920B013 or from a day 7 post–symptom onset NP swab sample from subject 1920B011. Subject 1920B004 had detectable virus on an NP swab sample from 4 days post–symptom onset by qPCR; however, we were unable to sequence virus from the small amount of viral RNA present in that sample. Only one sample, an NP swab obtained from subject 1920B007 three days after symptoms began, had sufficient viral RNA to perform sequencing. We detected no nonsynonymous (amino acid-changing) mutations in any epitopes targeted by IBV-specific T-cell populations in the lower airways of that subject at the hyperacute time point (Fig. S3), consistent with the phylogenetic conservation of these viral proteins.

### IBV-specific CD8^+^ T cells in the lower airways exhibit a unique transcriptional phenotype

**Figure 3:**
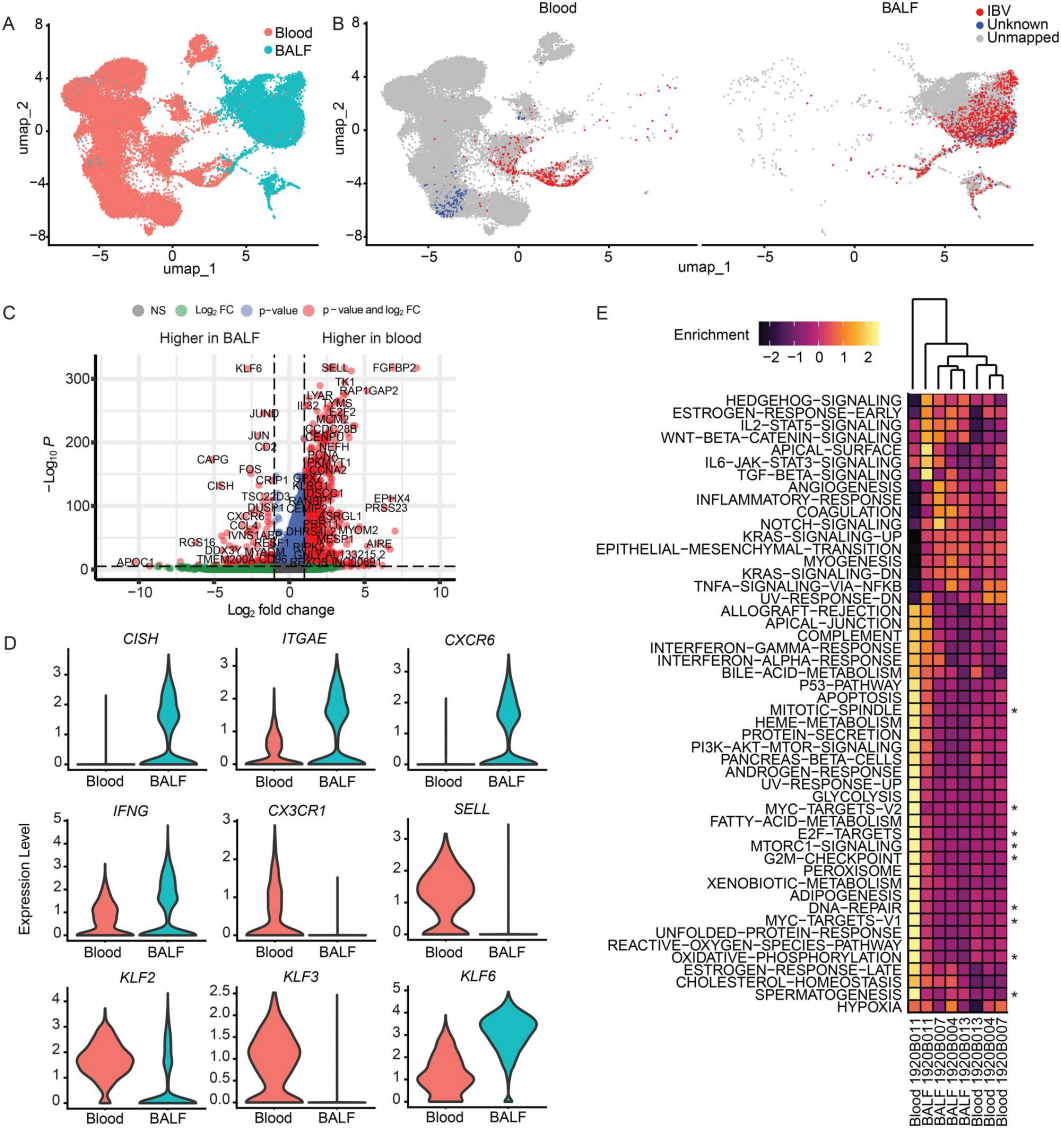
Human IBV-specific CD8^+^ T cells exhibit distinct transcriptional profiles in blood and BALF. (A) UMAP plot of all cells containing both a full-length TCRα and TCRβ sequence. (B) UMAP plot of cells with full-length TCRα and TCRβ sequence from the blood (left panel) and BALF (right panel) highlighting reverse epitope–mapped IBV-specific TCR-containing cells in red and cells with TCR of unclear specificity from reverse epitope mapping in blue. Cells with TCRs that were not reverse epitope mapped are in gray. (C) Volcano plot of all differentially expressed genes from IBV-specific cells in BALF (left) and blood (right). (D) Expression of indicated selected genes that were significantly different after adjustment for multiple comparisons between IBV-specific T cells found in either the blood or BALF. (E) Heatmap of expression level of Hallmark gene sets in IBV-specific T cells found in the indicated samples and subjects. Samples and subjects are hierarchically clustered according to the scaled enrichment score of the indicated gene sets. The 9 gene sets significantly different between blood and BALF after correcting for multiple comparisons are denoted with an asterisk (*).

To characterize the transcriptional phenotypes of the IBV-specific T cells, we performed universal manifold approximation and projection (UMAP) dimensionality reduction of transcriptional profiles from a total of 69,150 cells, including 33,937 cells found in the BALF and 35,213 sorted blood non-naïve CD8^+^ T cells. This analysis revealed multiple distinct transcriptional clusters informatically classified as CD8^+^ T cells and airway-resident subsets (Fig. S4A, B). We selectively analyzed the subset of this larger dataset that included all CD8^+^ T cells with a paired αβ TCR. Blood (*n* = 28,625) and BALF (*n* = 12,816) CD8^+^ T cells analyzed in this way exhibited multiple distinct clusters in UMAP dimensionality reduction analysis (Fig. S4C). These clusters exhibited very little overlap between blood and BALF (Fig. 3A). Interestingly, IBV-specific clonotypes identified by reverse epitope mapping co-localized together on the UMAP in both the blood and BALF (Fig. 3B), consistent with shared phenotypes for these antigen-specific cells within their specific tissue compartments.

Comparison of 834 IBV-specific CD8^+^ T cells in blood to 4171 IBV-specific CD8^+^ T cells in BALF revealed 7806 differentially expressed genes between the 2 tissue compartments after correcting for multiple comparisons (Fig. 3C, full dataset included as File S1). The top significantly different genes were dominated by tissue residency genes including *SELL*, *KLF3*, *CX3CR1*, and *S1PR1* (File S1).^1^^,^^2^^,^^21^^,^^22^ We found almost exclusive expression of the tissue residency gene *CXCR6* and much higher expression of the tissue residency gene *ITGAE* in IBV-specific BALF cells (Fig. 3D). Conversely, circulating and patrolling memory T-cell genes including *SELL* and *CX3CR1* were almost exclusively expressed in blood IBV-specific CD8^+^ T cells compared to those from the BALF (Fig. 3D). Transcription factors known to be associated with tissue residency establishment *KLF2* and *KLF3* were substantially upregulated in circulating IBV-specific CD8^+^ T cells, whereas the transcription factor *KLF6*, a gene with limited descriptions of relevance in tissue residency, and the *CISH* gene, a member of the cytokine-induced STAT inhibitor family, were upregulated in BALF IBV-specific CD8^+^ T cells (Fig. 3D).

We next performed gene set enrichment analysis with hierarchical clustering of significantly different gene sets between the individual blood and BALF samples. This revealed evidence of T-cell activation and cell cycle gene upregulation in the IBV-specific CD8^+^ T cells from the blood and, to a lesser degree, the BALF of the single subject with severe disease (Fig. 3E). Despite the distinctive phenotype of the severe subject’s IBV-specific blood CD8^+^ T-cell population in this analysis, the remaining blood and BALF samples co-segregated in the hierarchical clustering analysis, supporting our hypothesis that unique and consistent phenotypes exist in IBV-specific CD8^+^ T cells found in each of the 2 tissues (Fig. 3E).

### IBV-specific CD8^+^ T cells in the lower airways express more antiviral cytokines and fewer cytolytic proteins

**Figure 4:**
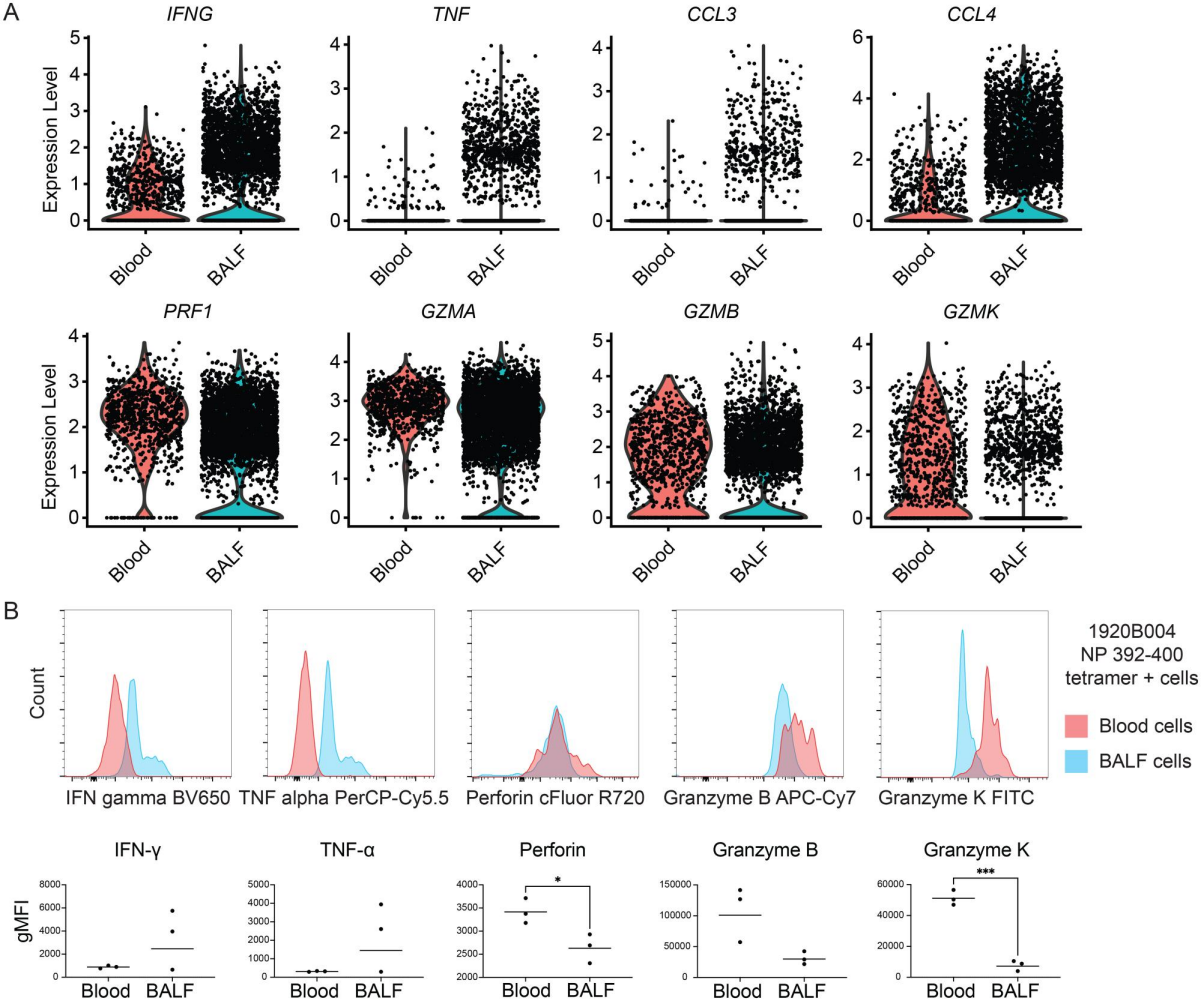
Human IBV-specific CD8^+^ T cells in the BALF express more antiviral cytokines and IBV-specific cells in blood express more cytotoxic effectors. (A) Expression level of each indicated gene in all IBV-specific T cells found in the indicated subjects and samples. All transcriptional differences are significant after adjustment for multiple comparisons. Dots were overlaid to demonstrate single-cell expression differences even when the majority of cells did not express a gene, as illustrated by the underlying violin plot. (B) Intracellular expression of indicated proteins in tetramer-positive CD8^+^ T cells from blood or BALF. These are the same tetramer responses plotted in Fig. 2. Top panels provide representative histograms of data from the NP 392-400 response in subject 1920B004. Three data points in the bottom panels include the NP 392-400 response in 1920B004, the NP 92-99 response in 1920B007, and the M1 119-126 response in 1920B013. Geometric mean fluorescent intensity (gMFI) is plotted with a line demarcating the geometric mean of the indicated group. *P < 0.05, ***P < 0.005 by Welch’s t test.

Following activation through the TCR, CD8^+^ T cells mediate their antiviral function through 2 broad pathways: antiviral cytokine expression and/or cytotoxic molecule expression. Animal models suggest that antiviral cytokines, rather than cytotoxic molecules, are the principal method whereby influenza-specific lung tissue-resident CD8^+^ T cells exert their antiviral effects.^23^ We next evaluated for differences in the expression of key mediators of these 2 functional pathways in IBV-specific CD8^+^ T cells from the 2 tissues. Antiviral cytokine genes including *IFNG*, *TNF*, *CCL3*, and *CCL4* were expressed at significantly higher levels in BALF IBV-specific CD8^+^ T cells compared to blood IBV-specific CD8^+^ T cells (Fig 4A). Cytotoxicity genes including perforin (*PRF1*), granzyme A (*GZMA*), granzyme B (*GZMB*), and granzyme K (*GZMK*) were expressed at significantly higher levels in blood IBV-specific CD8^+^ T cells compared to BALF IBV-specific CD8^+^ T cells (Fig. 4A). We confirmed this pattern of higher cytotoxic protein expression in blood IBV-specific T cells and higher antiviral cytokine expression in BALF IBV-specific T cells at the protein level by analyzing intracellular IFN-γ, TNF-α, perforin, granzyme B, and granzyme K in unstimulated IBV-tetramer positive cells from remaining BALF and blood samples of the 3 outpatient subjects using FACS (Fig. 4B). This analysis demonstrated significantly increased perforin and granzyme K protein in IBV-specific CD8^+^ T cells in the blood compared with matched IBV-specific CD8^+^ T cells in BALF.

## Discussion

Our data show that human adults mount a robust and clonally expanded CD8^+^ T-cell response against seasonal influenza in the lower airways following acute infection. This response is remarkably focused, with single epitope-specific responses composing 21% to 70% of the entire BALF CD8^+^ T-cell repertoire. The majority of clonally expanded TCRs in the BALF target phylogenetically conserved internal influenza viral proteins. Lower airway influenza-specific CD8^+^ T cells exhibit a tissue-resident phenotype and express high levels of antiviral cytokines with constrained expression of cytotoxic effector molecules. This unique functional phenotype, similar to that observed and characterized in animal models,^23^ presumably serves to minimize tissue damage in the delicate lower airway tissue compartment, where substantial cytotoxicity of infected and bystander cells could impair critical gas exchange. In addition, IFN-γ expression by human lung tissue-resident CD8^+^ T cells has been shown to facilitate very rapid antiviral responses that restrict influenza replication in ex vivo cultured human lung airway epithelial cells,^24^ underscoring the importance of the high IFN-γ expression we observe in lower airway IBV-specific CD8^+^ T cells following acute infection.

Clinical disease caused by IBV is a major public health issue and causes significant excess hospitalizations and mortality during IBV-predominant influenza seasons.^25^ The severity of disease in individuals hospitalized with IBV illness is statistically similar to illness caused by influenza A infection.^26^ Despite the public health implications and need for improved IBV coverage in vaccines, knowledge of human T-cell responses directed against IBV is extremely limited. Only 10% of influenza-specific CD8^+^ T-cell epitopes listed in the publicly available Immune Epitope Database represent uniquely described IBV-specific responses (39 unique IBV-specific epitopes out of 382 total influenza-specific CD8^+^ T-cell epitopes; iedb.org, search date September 29, 2025). Much of the published IBV-specific T-cell literature focuses on responses that are cross-reactive with influenza A virus.^19^^,^^27^ Our present work describes 10 novel IBV-specific CD8^+^ T-cell epitopes and expands the currently known number of IBV-specific epitopes by >20%. More importantly, we show that comprehensive profiling of the most dominant influenza-specific CD8^+^ T-cell responses in the lower airways can be accomplished with a sequencing-first reverse epitope mapping approach. This approach can be applied in larger cohorts of HLA diverse populations infected with various strains of influenza to characterize and prioritize T-cell epitope-rich antigens for novel T cell–focused vaccine candidates.

Our observations following acute IBV infection are distinct from recent results reporting minimal clonal expansion of SARS-CoV-2–specific CD8^+^ T cells in the lower airways during primary infection^28^ and suggest that repeated influenza infections during a lifetime may lead to distinct dominant clonal memory tissue-resident influenza-specific CD8^+^ T-cell populations focused on conserved viral epitopes, where only a handful of clonal TCRs represent the bulk of the response in adults. Alternatively, influenza-specific CD8^+^ T cells in the lower airways may exhibit unique activation and recruitment dynamics when compared with SARS-CoV-2–specific T cells that favor oligoclonal expansion. Indeed, repeated exposure to SARS-CoV-2 is relatively novel to the human population, and future examination of SARS-CoV-2–specific CD8^+^ T cells in the lower airways may more closely resemble our present results.

We were unable to detect nonsynonymous variation in the regions of the virus targeted by the strongly immunodominant lower airway CD8^+^ T-cell response when evaluating sequenced autologous IBV. Our experiment was limited by the low amount of ongoing viral replication detected in available upper and lower airway samples from our cohort. We hypothesize that the very low number of strongly immunodominant influenza-specific TCR clones we observe in the lower airways may have implications for influenza viral evolution under such focused immune pressure at the population level,^29^ despite our lack of detection of variation in response to this immune bottleneck here. Furthermore, limited memory influenza-specific TCR diversity in the lower airways has important implications for ongoing efforts to develop influenza vaccines that boost T-cell responses where more diverse clonal responses may provide more cross-recognition of viral variants.

Our work does have limitations. First, we comprehensively characterize lower airway T-cell responses from only 3 outpatient subjects and one subject with severe illness. While these individuals were relatively HLA diverse, the small sample size may not capture variation seen in individuals with other HLA alleles that may exhibit airway immunodominance hierarchies different from the restricted pattern we observe here. Furthermore, while the tissue-resident and functional phenotypes we observe at the transcriptional and protein level are consistent in all 4 subjects, it is possible that breakdown of the blood/lower airway interface during more severe disease may diminish such a compartmentalized influenza-specific T-cell response. Despite these limitations, we show that more than half of the total number of T cells in the lower airways can be influenza-specific during acute infection. These responding cells appear to exhibit a functionally distinct phenotype when compared with circulating influenza-specific cells. In addition, the most immunodominant responses in the lower airways are not always reflected in the circulating T-cell response. Our work underscores the importance of observing human antigen-specific T-cell responses at the site of infection to unravel human T-cell biology.

## Supplementary Material

vkag068_Supplementary_Data

## Data Availability

Single-cell RNA sequencing data are available in the Gene Expression Omnibus database under accession number GSE307746.
