# Supplementary material for: Highly focused human CD8+ T-cell response in the lower airways during acute influenza infection
Source: J Immunol. 2026 May 19;215(5):vkag068. doi: 10.1093/jimmun/vkag068 (PMC13183717; doi:10.1093/jimmun/vkag068)
Supplement: vkag068_Supplementary_Data [file vkag068_supplementary_data.zip › Figure S4.pdf]

Supplemental Figure 4

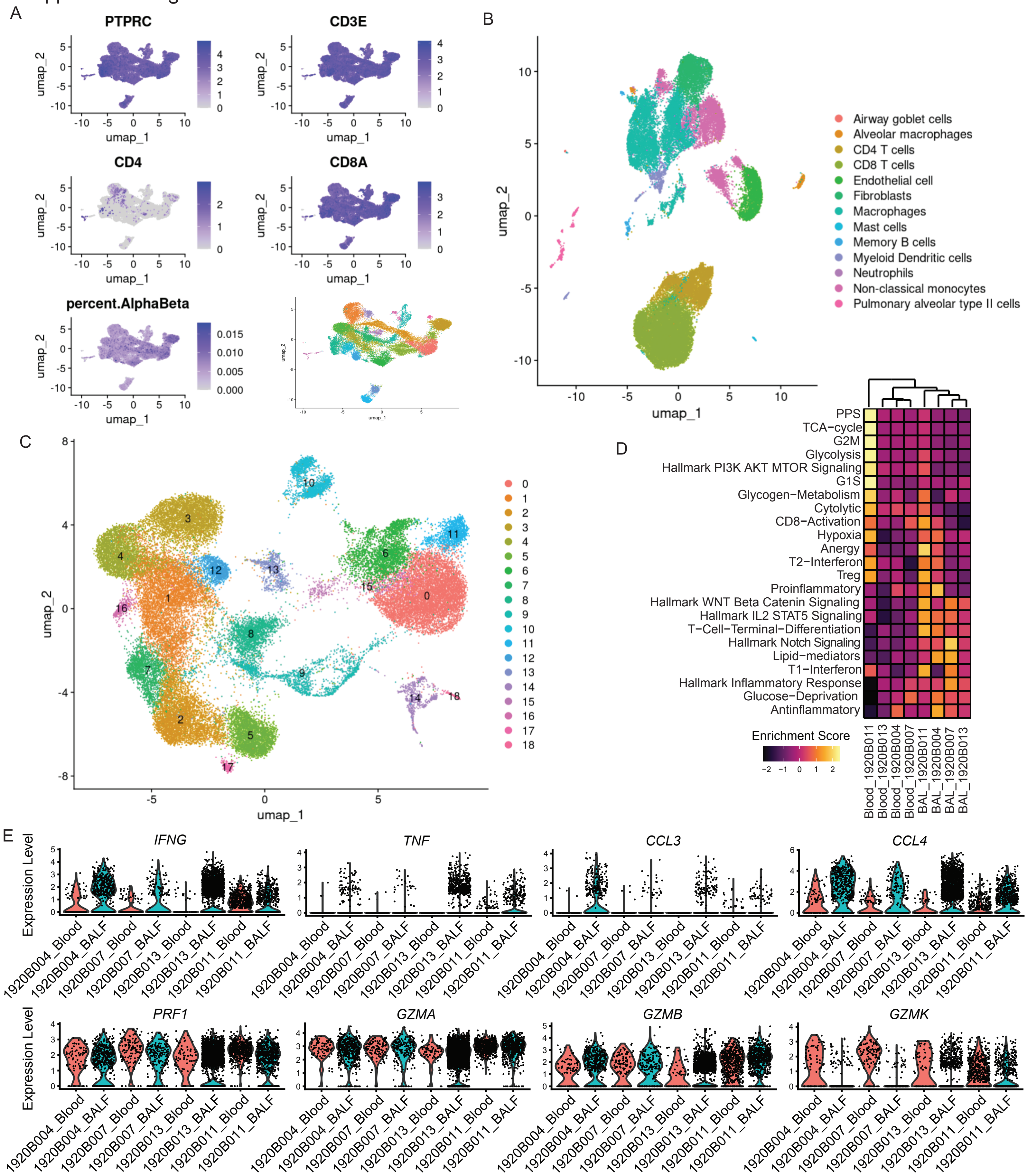

Supplemental Figure 4. Single-cell RNAseq of blood and BALF samples. **A)** UMAP plots of FACS-sorted non-naïve CD8$^+$ T cells from blood. Feature plots demonstrate that typical CD8 markers overlap distinct transcriptional clusters (bottom right). Percent.AlphaBeta corresponds to the percentage of expression owed to αβ TCR gene segments. **B)** UMAP plot of BALF cells. CD4$^+$ and CD8$^+$ T cells were annotated using the same markers as in **A)**. All other cell types were inferred using SCType. **C)** UMAP plot of all cells with detected paired αβ TCRs. This plot corresponds to **Figure 3A**. **D)** Heatmap of expression level of select immunologically relevant gene sets in IBV-specific T cells found in the indicated samples and subjects. Samples and subjects are hierarchically clustered according to the scaled enrichment score of the indicated gene sets. **E)** Expression level of each indicated gene in all IBV-specific T cells found in the indicated subjects and samples.
