# Supplementary material for: Highly focused human CD8+ T-cell response in the lower airways during acute influenza infection
Source: J Immunol. 2026 May 19;215(5):vkag068. doi: 10.1093/jimmun/vkag068 (PMC13183717; doi:10.1093/jimmun/vkag068)
Supplement: vkag068_Supplementary_Data [file vkag068_supplementary_data.zip › Figure S1.pdf]

Supplemental Figure 1

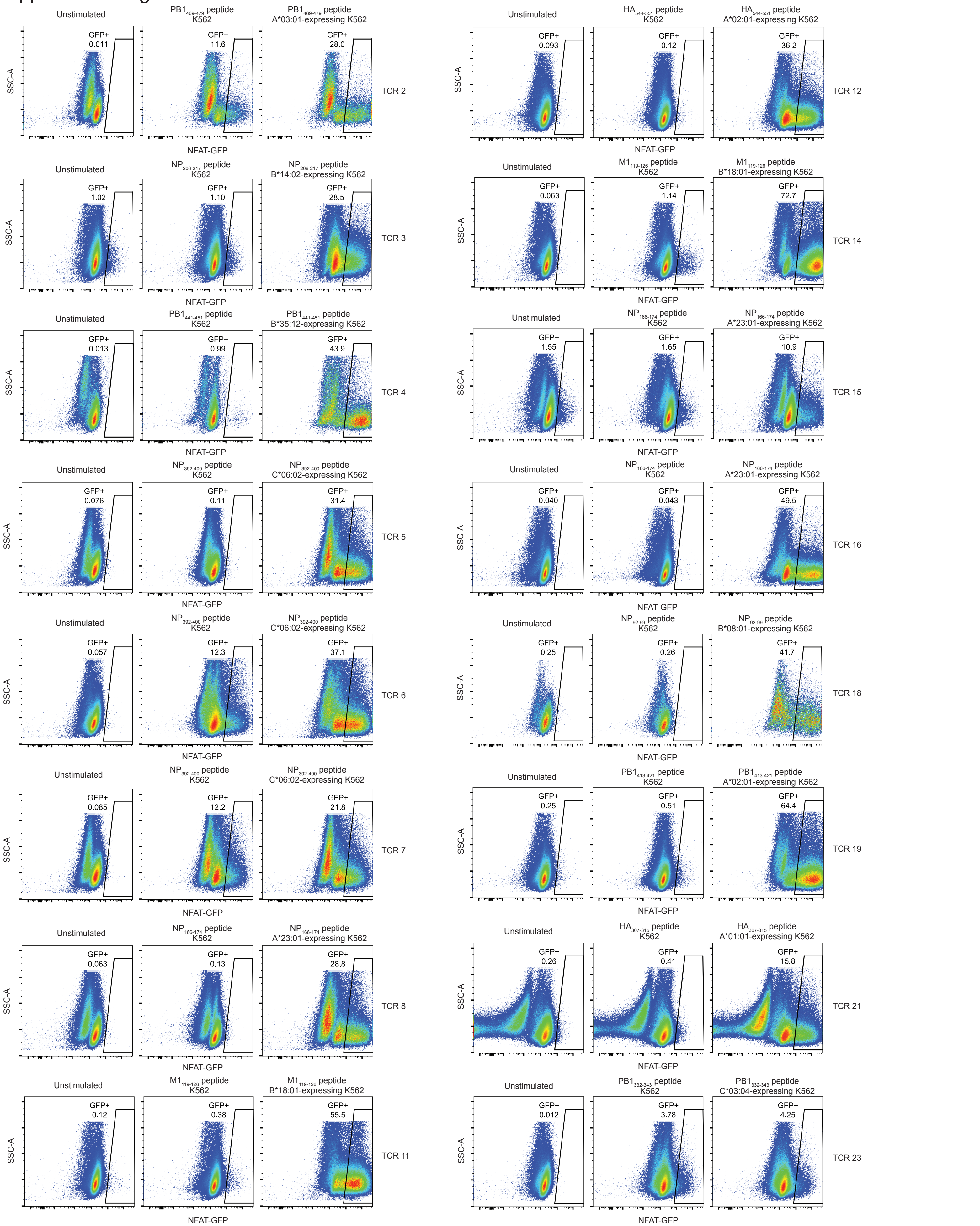

Supplemental Figure 1. Nuclear factor of activated T cells (NFAT)-green fluorescent protein (GFP) reporter CD8+ Jurkat T cell line reverse epitope mapping results. Each set of panels represents the indicated transduced TCR reporter Jurkat T cell line incubated without any peptide (left panel), incubated with the mapped IBV peptide in the presence of K562 cells (middle panel), and incubated with the mapped IBV peptide in the presence of K562 cells transduced with the indicated human HLA class I allele. Each HLA-restriction experiment was performed at least twice and the transduced TCR sequence was confirmed by sequencing genomic DNA of the responding Jurkat line following the experiment.
