# Supplementary material for: Highly focused human CD8+ T-cell response in the lower airways during acute influenza infection
Source: J Immunol. 2026 May 19;215(5):vkag068. doi: 10.1093/jimmun/vkag068 (PMC13183717; doi:10.1093/jimmun/vkag068)
Supplement: vkag068_Supplementary_Data [file vkag068_supplementary_data.zip › Figure S2.pdf]

Supplemental Figure 2

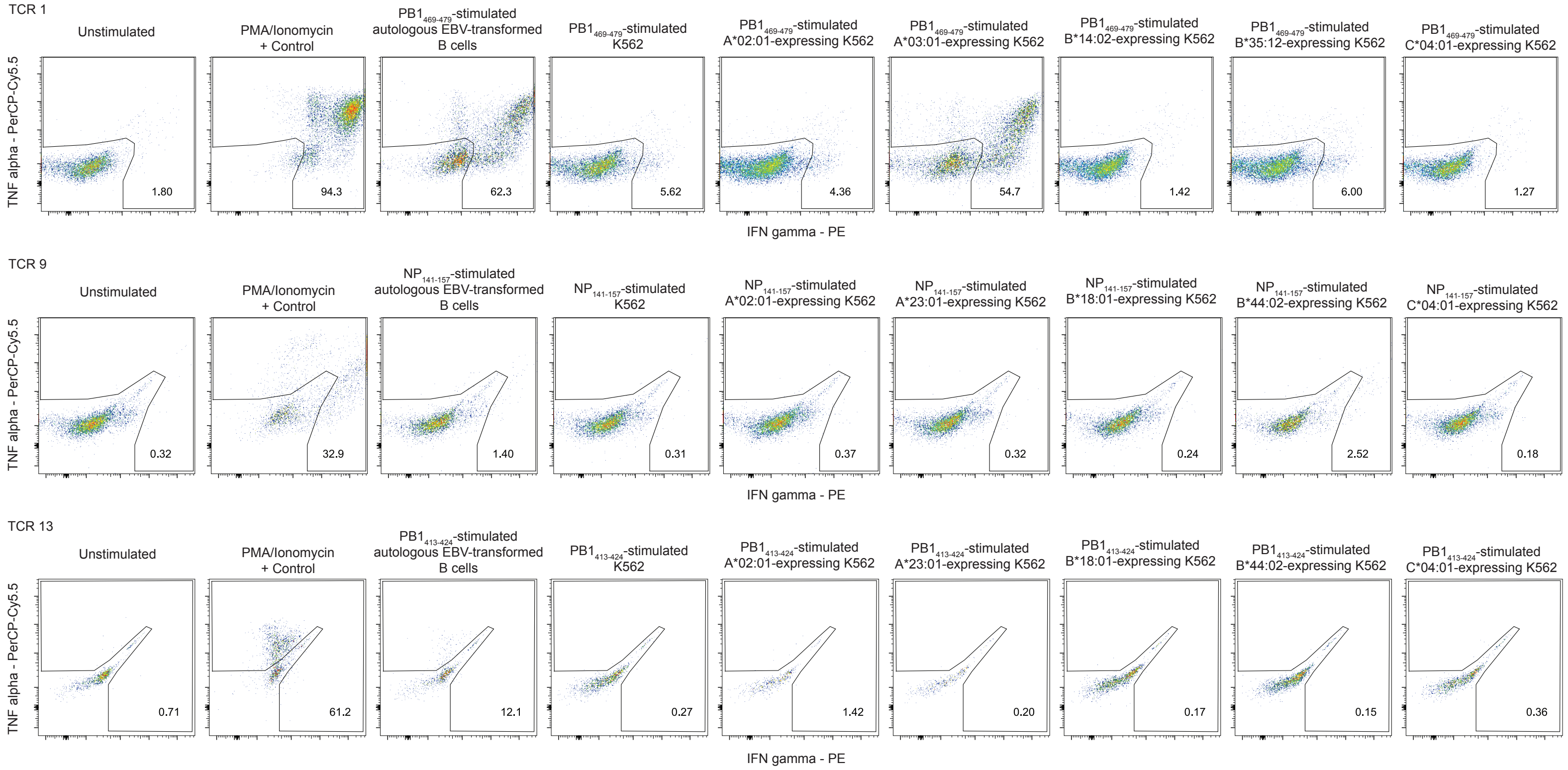

Supplemental Figure 2. TCR transduced primary human cell line reverse epitope mapping results. Primary human $CD8^+$ T cells retrovirally-transduced with the indicated experimental TCR were incubated with the specified stimulus for 16 hours in the presence of golgi inhibitors prior to surface and intracellular staining. $CD8^+$ T cells expressing the engineered murine TCR beta constant chain were gated and intracellular interferon (IFN) gamma and tumor necrosis factor (TNF) alpha were measured for each permutation. Each experiment was performed at least twice using two or more independently generated primary human $CD8^+$ T cell lines made from a sequencing-confirmed retrovirus stock.
