# Supplementary material for: Highly focused human CD8+ T-cell response in the lower airways during acute influenza infection
Source: J Immunol. 2026 May 19;215(5):vkag068. doi: 10.1093/jimmun/vkag068 (PMC13183717; doi:10.1093/jimmun/vkag068)
Supplement: vkag068_Supplementary_Data [file vkag068_supplementary_data.zip › Figure S3.pdf]

Supplemental Figure 3

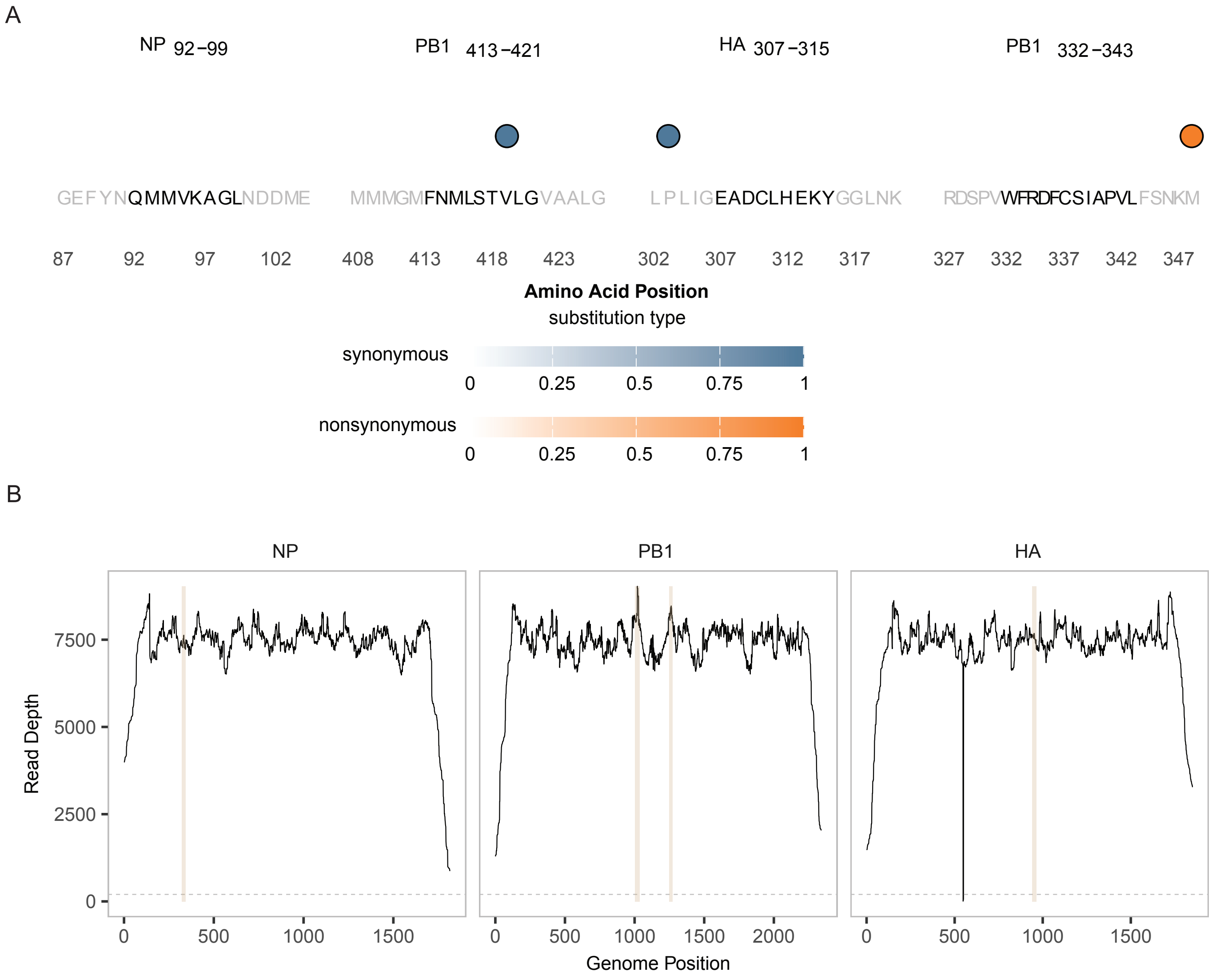

Supplemental Figure 3. Autologous influenza virus sequencing from subject 1920B007 day 3 post-symptom onset NP swab sample. All detected mutations in the NP swab sample were fixed (found in > 98% of viral quasispecies) and many were observed in contemporary circulating variants suggesting they represent the infecting strain. **A)** Dots above amino acid sequences denote codon locations of detected intra-host single nucleotide variants compared to reference 2019-2020 vaccine strain B/Iowa/06/2017/Victoria (blue, synonymous; orange, nonsynonymous) and are shaded by substitution frequency. Epitope regions are noted in black text. Flanking regions are shown in gray text. **B)** Sequencing depth across IBV segments containing IBV epitopes for 1920B007 day 3 NP swab sample. Epitope regions are highlighted in tan. Gray dotted line represents the sequencing depth cutoff of 200 required for calling variants. Vertical black line in HA indicates lack of coverage caused by an apparent in-frame deletion of HA nucleotides 548-550.
